# Comparative Analysis of CpG Sites and Islands Distributed in Mitochondrial DNA of Model Organisms

**DOI:** 10.3390/ani10040665

**Published:** 2020-04-11

**Authors:** Krzysztof Kowal, Angelika Tkaczyk, Tomasz Ząbek, Mariusz Pierzchała, Brygida Ślaska

**Affiliations:** 1 Institute of Biological Bases of Animal Production, Faculty of Animal Sciences and Bioeconomy, University of Life Sciences in Lublin, Akademicka 13, 20-950 Lublin, Poland; krzysztof.kowal@up.lublin.pl (K.K.); angelika.tkaczyk92@gmail.com (A.T.); 2 Department of Animal Genomics and Molecular Biology, National Research Institute of Animal Production, Krakowska 1, 32-083 Balice, Poland; t.zabek@izoo.krakow.pl; 3 Department of Genomics and Biodiversity, Institute of Genetics and Animal Breeding, Polish Academy of Sciences, Postepu 36a Str., 05-552 Jastrzebiec, Poland; m.pierzchala@ighz.pl

**Keywords:** CpG sites, mtDNA, model organisms

## Abstract

**Simple Summary:**

In recent years, the existence of methylation of mammalian mitochondrial DNA (mtDNA) has been discussed. The current state of knowledge indicates that mtDNA is poorly methylated; in fact, it only accounts for 2–8% methylated sites and its pattern is unknown. The lack of comprehensive information on the mtDNA methylation pattern prompted us to investigate the distribution of guanine-cytosine-rich sequences (CpG) in different animal species. The aim of the study was to determine the localization of CpG sites and islands in mtDNA of model organisms. The CpG sites and islands found in vertebrates and invertebrates indicate a diversified pattern of CpG distribution. Generally, the number of observed CpG sites of the mitochondrial genome was higher in the analysed vertebrates than in the invertebrates. However, there was no relationship between the frequency of the CpG sites in the mitochondrial genome and the complexity of the analysed organism. The distribution of the CpG sites for transfer RNA (tRNA) coding genes was usually cumulated in a larger CpG region in the vertebrates.

**Abstract:**

The information about mtDNA methylation is still limited, thus epigenetic modification remains unclear. The lack of comprehensive information on the comparative epigenomics of mtDNA prompts comprehensive investigations of the epigenomic modification of mtDNA in different species. This is the first study in which the theoretical CpG localization in the mtDNA reference sequences from various species (12) was compared. The aim of the study was to determine the localization of CpG sites and islands in mtDNA of model organisms and to compare their distribution. The results are suitable for further investigations of mtDNA methylation. The analysis involved both strands of mtDNA sequences of animal model organisms representing different taxonomic groups of invertebrates and vertebrates. For each sequence, such parameters as the number, length, and localization of CpG islands were determined with the use of EMBOSS (European Molecular Biology Open Software Suite) software. The number of CpG sites for each sequence was indicated using the newcpgseek algorithm. The results showed that methylation of mtDNA in the analysed species involved mitochondrial gene expression. Our analyses showed that the CpG sites were commonly present in genomic regions including the D-loop, *CYTB*, *ND6*, *ND5*, *ND4*, *ND3*, *ND2*, *ND1*, *COX3*, *COX2*, *COX1*, *ATP6*, *16s rRNA*, and *12s rRNA*. The CpG distribution in animals from different species was diversified. Generally, the number of observed CpG sites of the mitochondrial genome was higher in the vertebrates than in the invertebrates. However, there was no relationship between the frequency of the CpG sites in the mitochondrial genome and the complexity of the analysed organisms. Interestingly, the distribution of the CpG sites for tRNA coding genes was usually cumulated in a larger CpG region in vertebrates. This paper may be a starting point for further research, since the collected information indicates possible methylation regions localized in mtDNA among different species including invertebrates and vertebrates.

## 1. Introduction

Nearly all animal mitochondrial genomes are about 16. 5 kbp (kilo base pairs) in length, whereas plant mitochondrial genomes range between 200 and 2000 kbp [1]. Generally, the mammalian mitochondrial genome is a circular double-stranded DNA (dsDNA) molecule containing 13 protein-coding genes, 22 transfer RNAs (tRNAs), two ribosomal RNAs (rRNAs) genes, and one non-coding control region (D-loop region) [2]. The exception is the mtDNA genome of *Caenorhabditis elegans*, which lacks the *ATP8* gene [3] and the non-coding AT region. The non-coding region of mtDNA contains an origin of replication and three promoters: one for the light strand (LSP) and two for the heavy strand (HSP1 and HSP2). Transcription begins from promoters: LSP and HSP2 encode 13 protein-coding genes involved in the oxidative phosphorylation (OXPHOS) and 22 tRNAs, whereas HSP1 generates a short transcript containing rRNA genes [4]. MtDNA is packed into structures called nucleoids or mitochromosomes. The major part of the nucleoid constitutes transcription factor A (TFAM), which contributes to mtDNA packing. Hence, any alterations in the TFAM content influence the mitochromosome and, consequently, mtDNA is exposed to DNA methyltransferases (DNMTs) [5].

The activity of methyltransferase (DNMT) was first detected in mitochondria isolated from loach embryos in 1970s [6]. Next, 5-methylcytosine (m^5^C) was found in beef heart mitochondria [7]. In 1977, the specificity of nuclear and mitochondrial DNMT was demonstrated. The mitochondrial and nuclear enzymes are specific to monopyrimidines and di- and tripyrimidines, respectively [8]. Reis and Goldstein [9] and Pollack et al. [10] conducted a study on mitochondria from human and mouse fibroblasts. Their results indicated that methylation in mtDNA occurred with a frequency of 2–5% only in CpG dinucleotides. Currently, the methylation frequency in mtDNA ranges from 2 to 8%, but its pattern is unknown [11]. Scientists found that nearly 25% of all methylations identified in embryonic stem cells were non-CpG methylations (CpA, CpT, and CpC). In normal somatic cells, the non-CpG methylation level is relatively low, with enrichment mainly in the coding regions of active genes [12,13]. 

Little is known about mitochondrial epigenetic modifications, as studies on mtDNA methylation are not as common as studies on the methylation of nDNA. So far, it has been observed that hypomethylation may occur in mtDNA methylation [4,14,15]. During methylation, DNA undergoes covalent modification, usually at cytosine residues within CpG dinucleotides, and is catalysed by DNA methyltransferase (DNMT) in the presence of the methyl donor S-adenosyl-L-methionine (SAM) [16]. Clusters of CpG sites form GC-rich islands that have a CpG located approximately every 10 base pairs [17,18].

In recent years, the methylation of mammalian mtDNA has gained interest. There are papers rejecting the existence of mtDNA methylation [19]. For instance, Hong et al. [11] used the bisulfite genomic sequencing method to determine CpG methylation in a human colon cancer cell line and primary human cells. Additionally, next-generation sequencing was used for total DNA. As a result, no CpG methylation was found in mtDNA [11]. In turn, Bellizzi et al. [20] detected methylated cytosines in the D-loop region of mtDNA isolated from blood and cultured cells from humans and mice. To address the controversy of the existence of mtDNA methylation, an interesting study on the methylation of the D-loop was conducted by Liu et al. [21]. They confirmed the existence of methylation with varying frequency in different human tissues. The analysis of 6 CpG sites in human blood samples indicated that the methylation level varied from 2% to 34% but was almost undetectable in saliva. Generally, the estimated average frequency of mtDNA methylation was lower than 2%. Moreover, it was found that the form of mtDNA had an impact on its level. It has been found that the circular structure affects the bisulfite conversion efficiency, hence mtDNA methylation is overestimated [21]. The evidence supporting mtDNA methylation is related to mtDNA abnormalities including amyotrophic lateral sclerosis [22], Down’s syndrome (DS) [14], glioblastoma [23], or nonalcoholic fatty liver disease [24]. For instance, cultured amniocytes from DS patients showed TFAM downregulation [5]. In turn, Infantino et al. [14] detected hypomethylation in DS cells in which the mtDNA content was increased.

Despite new evidence, the information about mtDNA methylation is still limited. Therefore, this epigenetic modification is a controversial issue. The lack of comprehensive information on the comparative epigenomics of mtDNA suggests that there is a need to conduct comprehensive investigations of the epigenomic modification of mtDNA in different species. The aim of the study was to determine the localization of CpG sites and islands in mtDNA of model organisms and to compare its distribution. The results are suitable for further investigations of mtDNA methylation.

## 2. Materials and Methods

Reference sequences of twelve animal mtDNAs obtained from GenBank were analysed. The study was carried out on sequences of animal model organisms representing different taxonomic groups of invertebrates and vertebrates (Table 1).

In order to analyse both strands of mtDNA, sequences from GenBank were rewritten in the EMBOSS revseq algorithm to obtain complementary sequences representing the H-strand of mtDNA [25]. Regions with frequency of CG dinucleotides that were higher than expected were identified in each of the 24 analysed sequences from the 12 species. Two EMBOSS algorithms were used. The Cpgplot uses a sliding window within which the observed/expected ratio of CpG is calculated [26]. For a sequence region reported as a CpG island, the following constraints were established: the observed/expected ratio >0.6, %C + %G > 50%, and the sequence length should exceed 200 bp. The newcpgseek uses a running sum calculated from all positions in the sequence rather than a window to produce a score. If there is a missing CG dinucleotide at a position, the score is decremented; if there is a CG dinucleotide, the score is incremented by a constant (user-defined) value. When the score for a region in the sequence is higher than the threshold (17 at the moment), a putative island is declared. Sequence regions scoring above the threshold are searched for recursively. This method overpredicts islands but finds smaller ones around primary exons. The newcpgseek displays the actual CpG count, the %C + %G sum, and the observed/expected ratio in a region where the score is above the threshold [25]. For each sequence, such parameters as the number, length, and localization of CpG islands were determined. Using the newcpgseek algorithm, the number of the CpG sites for each sequence was indicated.

## 3. Results

### 3.1. CpG Islands in mtDNA

The positions of the CpG islands in the mtDNA of 12 organisms are presented in Table 2 and Table 3. There were no CpG islands on the strands of the mtDNA genomes of *Caenorhabditis elegans*, *Daphnia magna*, and *Drosophila melanogaster*, i.e. all invertebrates analysed in the study. In the analysed animal models, the length of the CpG islands varied from 202 bp to 313 bp in the L-strand and from 200 bp to 632 bp in the H-strand. The results of the L-strand showed that one CpG island was located in the *COX2* gene (*Homo sapiens* and *Gallus gallus*), and two CpG islands were found in the sequence from *Danio rerio* (Table 2). The longest CpG island among all the tested animal models was detected in the canine mtDNA genome located in the D-loop region in the position of the VNTR: 5′-GTACACGT(A/G)C-′3 region.

The present study showed an increased number of CpG islands on the H-strand of mtDNA, compared to the L-strand (Table 2, Table 3). It is worth noting that the mtDNA of *Gallus gallus* (7), *Crocodylus porosus* (4), and *Homo sapiens (4)* had the highest numbers of CpG islands. CpG islands were found frequently in genomic regions covering loci of *12s rRNA* (71%), *CYTB* (43%), *ND5* (57%), and *COX1* (43%). Interestingly, two CpG islands were observed in the *12s rRNA* and *ND5* genes from the mtDNA genome of *Crocodylus porosus* and the *COX1* gene from *Gallus gallus* (Table 3). Moreover, only in the *Danio rerio* genome was the CpG island located in tRNA-coding genes, which were also encoded on the L strand. The analysis of *Canis lupus familiaris* mtDNA showed the presence two CpG islands on both strands occupying the VNTR sequence in the D-loop region in the same localization (Table 3). 

### 3.2. Strongly Enriched CpG Regions in mtDNA

The distribution of CpG sites in the mtDNA genomes of the analysed animals and the total number of CpG sites for each animal model are presented in Table 4. The analyses showed that the CpG sites were commonly detected in genomic regions, including the D-loop, *CYTB*, *ND6*, *ND5*, *ND4*, *ND3*, *ND2*, *ND1*, *COX3*, *COX2*, *COX1*, *ATP6*, 16s rRNA, and 12s rRNA. The CpG distribution in animals varies. Generally, the number of the CpG sites of the mitochondrial genome was higher in the vertebrates than in the invertebrates. However, there was no relationship between the frequency of the CpG sites in the mitochondrial genome and the complexity of the analysed organism. CG-rich regions were mainly observed in genes encoding proteins or rRNA molecules; however, CpG dinucleotides were also found in non-coding sequences such as the *AT* region in the mtDNA of *Caenorhabditis elegans* and the *D-loop* in the vertebrates. Noteworthy, in some of the analysed species, e.g. *Homo sapiens*, *Pan troglodytes ellioti*, *Ambystoma mexicanum*, and *Crocodylus porosus*, CpG sites were found in intergenic areas (Table 5). The CpG sites were not commonly located in the tRNA coding genes. For example, no CpGs were observed in the locus of the *TRNQ* gene in any of the analysed species. It should be emphasized that CpG sites are distributed in a cluster overlapping many tRNA coding genes, such as *TRNW*, *TRNA*, *TRNN*, *TRNC*, and *TRNY* (Table 5). The in silico analysis revealed diverse distribution of the CpG sites in the replication origin region between both the species and the strands of the analysed vertebrates. Mammalian species share a structurally identifiable replication origin at a fixed mitochondrial genome location (between *TRNC* and *TRNN*), in contrast to avian and crocodilian species [27]. There were no CpG dinucleotides on the mtDNA of *Crocodylus porosus* and *Gallus gallus* in a location analogous to the region of the replication origin in the other vertebrates (Table 5).

## 4. Discussion

The methylation of the mtDNA is still a matter of debate [21]. The present study indicated plausible sites of methylation as epigenetic modification of mtDNA and demonstrated different levels of the distribution of CpG sites and islands in various animal model species. No CpG islands were detected in the invertebrates, whereas CpG sites were found in both the invertebrates and the vertebrates, but they occurred frequently in the more complex organisms. This is the first study presenting the theoretical CpG localization on both strands of the mtDNA reference sequences in various species. As demonstrated by available literature, *Caenorhabditis elegans* does not have a DNMT; hence, no methylation is detected [28]. However, invertebrates with very low or undetectable methylation of CpG, e.g. *Drosophila melanogaster* or *Caenorhabditis elegans*, are a minority, as reported by Suzuki et al. [17]. In most invertebrates, mosaic nDNA methylation takes place, but it is not clearly known whether it occurs in mtDNA [17]. A low level of methylation was observed in the case of essential genes, including *CYTB*, *COX1*, and 12s rRNA. Moreover, despite the lack of CpG islands in *Caenorhabditis elegans*, single CpG sites were observed in the non-coding AT-region (Table 4), which is located between the tRNA^ala^ and tRNA^pro^ genes previously described by Okimoto et al. [29]. The low occurrence of CpG sites and islands in the mtDNA genomes of invertebrates may be related to the different modes of epigenetic control of replication and expression, such as non-CpG (CpA, CpT, and CpC) methylation. The co-existence of non-CpG sites was also observed within nDNA in human specific cell types such as stem cells, oocytes, neurons, and glial cells [30]. Yet, most CpG sites were indicated in protein-coding genes and rRNA-coding genes. The occurrence of CG nucleotides might be correlated with the length of the sequence: the longer the sequence, the greater the likelihood of a multitude of CpG sites. It should be emphasized that the theoretical presence of CpG sites and islands in the mtDNA genes of the analysed animals does not indicate methylation of these genes. However, the possibility of OXPHOS gene methylation within specific cells of the analysed animal species should not be excluded in certain circumstances. The oxidative phosphorylation system (OXPHOS) is a biochemical pathway located in the mitochondrial inner membrane responsible for energy production, apoptosis, and cell differentiation [31]. A proper OXPHOS function is important for cellular homeostasis, tissue dynamics, and health status of individuals [32].

Another non-coding region is the D-loop, where CpG sites observed in the region may be related to its regulative function of mtDNA in terms of replication and expression. Liu et al. [21] found that DNA methylation took place in the main non-coding region, which contains regulatory regions for the heavy (HSP1/2) and light strands (LSP) and an initiation site for heavy strand replication. First, replication is initiated at a specific site on the H-strand (called O_H_). After replication of two-thirds of the H-strand, the replication of the L-strand starts and proceeds in the opposite direction [2]. Except for *Canis lupus familiaris*, a higher number of CpG sites were found on the H-strand of mtDNA (Table 4). 

It is worth noting that four genes i.e. *CYTB*, *COX1*, *ND1*, and 12s rRNA, were rich in CpG sites in all the analysed sequences. *CYTB* called cytochrome c reductase and *COX1* encoding cytochrome C oxidase subunit 1 belong to respiratory chain complexes III and IV. They are involved in the electron transport chain of mitochondrial oxidative phosphorylation (OXPHOS) and are essential for ATP synthesis [33]. Additionally, methylation observed in the sequence of *Homo sapiens* including *CYTB*, *COX1*, D-loop, and 12s rRNA has been reported by Liu et al. [21].

The LPS promoter is an important component of the non-coding region contributing to the expression of the OXPHOS complex I subunit ND6 [21]. The methylation of the *ND6* gene was reported in many studies [24,34,35]. For instance, Pirola et al. [24] analysed the methylation of *ND6*, *COX1*, and the D-loop region with the use of quantitative methylation specific-PCR in the context of non-alcoholic fatty liver disease in humans [24]. The authors found a significant association between the condition of non-alcoholic steatohepatitis (NASH) and the methylation of the *ND6* gene, which inversely correlated with ND6 transcription and protein expression in the liver affected by NASH [24]. The results reported in this paper showed the presence of CpG sites in the *ND6* gene in all the analysed vertebrates and the number of CpG sites varying from 3 to 22. Noteworthy, all NADH dehydrogenase subunits (ND1, ND2, ND3, ND4, ND5, ND6) were rich in CpG sites in the mtDNA of the different species (Table 4). However, the *ND3* and *ND6* genes were rich in CpG sites only in the vertebrates. The regulation of single subunits of NADH dehydrogenase is not completely understood. In the case of oxidative stress, DNMT is upregulated and suppresses the expression of the *ND6* gene through methylation. In turn, the downregulation of *ND6* contributes to upregulation of *ND1* [5]. ROS (reactive oxygen species) are targeted at mitochondria; hence, it has been proposed that the increased level of DNMT1 reflects adaption to oxidative stress. MTERF1 (mitochondrial terminator factor 1) probably interacts with m^5^C in CpG dinucleotides or with mtDNA and, consequently, DNMT1 is bound [5]. These results indicate that all the analysed sequences (with the exception of the L-strand of *Drosophila melanogaster*) have CpG sites in the *ND1* gene; however, the CpG distribution in the *ND6* gene is mainly limited to the H-strand of chordates. Moreover, the number of the CpG sites in *ND1* was higher (from 2 to 58) than in *ND6* (from 3 to 22), especially in *Latimeria chalumnae*, *Ambystoma mexicanum*, *Crocodylus porosus*, *Gallus gallus*, *Mus musculus*, *Canis lupus familiaris*, *Pan troglodytes ellioti*, and *Homo sapiens* (Table 4). 

The transcription of the H-strand of mtDNA starts at two initiation sites (H1, H2) within the control region. The produced transcript from H1 terminates at the 3′ end of the 16S rRNA gene and processes the two rRNAs and two tRNAs, whereas H2 terminates at the 5′ end of the 12S rRNA gene and generates a polycistronic molecule contributing to the mRNAs and most of the tRNAs encoded in the H strand [36]. The mitochondrial transcription termination factor (mTERF) has been associated with the H1 and H2 binding sites, but Martin et al. [36] evidenced only mTERF-bound by H1. The distribution of methylation of CpG sites in these regions may influence transcription regulation. Martin et al. [36] demonstrated that 12s rRNA is commonly methylated in the mitochondrial regions in animals. The CpG sites were widely distributed in the genomes of all the animals analysed in our study, but higher numbers were found in the vertebrates (Table 4). Methylation of the *16s rRNA* gene was recognized as an emerging resistance mechanism against aminoglycosides and was evidenced in microorganisms that are often multidrug resistant. Methylation of 16s rRNA disturbs translation [37,38]. The present results showed the presence of CpG-rich regions of 16s rRNA genes in both the invertebrates and vertebrates but not in the carnivores (*Canis lupus familiaris*) and primates (*Pan troglodytes ellioti* and *Homo sapiens*). This may be caused by the 1-methyladenosine (m^1^A) modification in 16S rRNA catalysed by tRNA methyltransferase 61B (TRMT61B) [39].

In the analysis of mtDNA methylation, several challenges that can affect the correct detection of the levels of mtDNA methylation have to be overcome. The first one is the high mtDNA copy number in cells; it naturally varies from hundreds to thousands of copies depending on the cell type. Application of super-resolution microscopy provides more details. Currently, the number of mtDNA molecules per nucleoid in human cells is estimated at 1.4 [40]. Another problem is the presence of nuclear mitochondrial sequences (Numts), first denoted as “NUMT” in the cat [41], which refer to a DNA segment transferred from mtDNA to nDNA. This phenomenon was observed in various eukaryotes including plants (*Arabiopsis thaliana*, *Oryza sativa*), invertebrates (*Caenorhabditis elegans*, *Drosophila melanogaster*), and vertebrates (*Mus musculus*, *Rattus norvegicus*, and *Homo sapiens*) [42]. Therefore, the issue whether low levels of CpG methylation occur in mtDNA or whether it is caused by contamination by methylated NUMTs is being questioned [43]. Moreover, the circular structure of mtDNA influences bisulfite conversion and causes overestimation of mtDNA methylation [21].

## 5. Conclusions

The theoretical study on the distribution of CpG sites and islands in the mitochondrial genome of twelve model animal species provides interesting information about the localization of CpG-rich regions that can be methylated in specific cells in certain conditions. The CpG methylation in mtDNA exerts an impact on various molecular processes, including replication, translation, and gene expression. Since methylation in mtDNA, in comparison to methylation in nDNA, is still not sufficiently understood, research in this area is advisable.

## Figures and Tables

**Table 1:** MtDNA reference sequences of analysed model organisms.

Organism	Accession Number of Reference Sequence *	Length of MtDNA (bp **)
*invertebrates*		
***Caenorhabditis elegans***	NC_001328.1	13,794
***Drosophila melanogaster***	NC_024511.2	19,524
***Daphnia magna***	NC_026914.1	14,948
*vertebrates*		
***Latimeria chalumnae***	NC_001804.1	16,407
***Danio rerio***	NC_002333.2	16,596
***Ambystoma mexicanum***	NC_005797.1	16,369
***Gallus gallus***	NC_040970.1	16,785
***Mus musculus***	NC_005089.1	16,299
***Canis lupus familiaris***	NC_002008.4	16,727
***Crocodylus porosus***	NC_008143.1	16,916
***Pan*** ***troglodytes ellioti***	KM679417.1	16,559
***Homo sapiens***	NC_012920.1	16,569

* NC, KM—nucleotide accession prefixes. ** bp—base pair.

**Table 2:** Positions of CpG islands in the mitochondrial genomes of the analysed animals on the light strand.

Organism	Genome Length (bp *)	% GC **	Positions of CpG Islands ***	Genome Region	Length of CpG Islands (bp)	Sum of C+G ****	%C + %G	Obs/Exp *****
***Danio rerio***	16,596	0.40	3281..3531	*16s rRNA*	251	126	50.20	0.95
			6205..6432	rep_origin, *TRNY*, *COX1*	228	120	52.63	0.91
***Gallus gallus***	16,785	0.46	8703..8925	*COX2*	223	118	52.91	0.97
***Canis lupus familiaris***	16,727	0.40	16,137..16,449	D-loop (VNTR)	313	170	54.31	2.71
***Pan troglodytes ellioti***	16,559	0.44	14,246..14,447	*CYTB*	202	103	50.99	1.27
***Homo sapiens***	16,569	0.44	7764..8036	*COX2*	273	137	50.18	1.13

* bp—base pair. ** guanine–cytosine (GC) base pairs. *** guanine-cytosine-rich regions (CpG islands). **** cytosine (C), guanine (G). ***** the observed/expected ratio.

**Table 3:** Positions of CpG islands in the mtDNA of the analysed animals on the H strand *.

Organism	Genome Length (bp **)	% GC ***	Start and Stop of MtDNA Sequence ****	MtDNA Region	Length of CpG Islands (bp) *****	Sum of C+G	%C + %G	Obs/Exp ******
***Danio rerio***	16,596	0.40	981..1180	*TRNI, 12s rRNA*	200	105	52.50	1.31
			6205..6432	*TRNN* *, *TRNY* *, *COX1*	228	120	52.63	1.17
***Latimeria chalumnae***	16,407	0.42	145..370	***12s rRNA***	**226**	**113**	**50.00**	**0.80**
***Crocodylus porosus***	16,916	0.43	51..311	***12s rRNA***	**261**	**133**	**50.96**	**1.61**
			12,371..12,699	***ND5***	**329**	**171**	**51.98**	**1.38**
***Gallus gallus***	16,785	0.46	1784..1992	***12s rRNA***	**209**	**108**	**51.57**	**1.23**
			6901..7108	*COX1*	208	111	53.37	1.25
			9456..9794	*ATP6*	339	174	51.33	1.25
			9920..10,551	*COX3*	632	323	51.11	0.99
			13,647..13,925	***ND5***	**279**	**143**	**51.25**	**1.23**
			14,984..15,210	***CYTB***	227	119	52.42	1.20
			16,297..16,508	*ND6*	212	110	51.89	0.99
***Canis lupus familiaris***	16,727	0.40	16,179..16,449	D-loop VNTR (16,130..16,430)	271	149	54.98	0.83
***Pan troglodytes ellioti***	16,559	0.44	2848..3136	*ND1*	289	146	50.52	1.26
			5572..5779	*COX1*	208	112	53.85	1.17
			12,379..12,642	***ND5***	**264**	**140**	**53.03**	**1.41**
			14,246..14,447	***CYTB***	202	103	50.99	1.27
***Homo sapiens***	16,569	0.44	1123..1352	***12s rRNA***	**230**	**115**	**50.00**	**1.15**
			3382..3717	*ND1*	336	178	52.98	1.26
			12,907..13,115	***ND5***	**209**	**109**	**52.15**	**1.29**
			14,804..15,044	***CYTB***	241	126	52.28	1.33

* genes in which CpG sites are frequently distributed among species were marked with bold font (genes encoded on the L strand). ** bp—base pair. *** guanine–cytosine (GC) base pairs. **** mitochondrial DNA (mtDNA). ***** guanine-cytosine-rich regions (CpG islands). ****** the observed/expected ratio.

**Table 4:** Distribution of CpG sites in the mtDNA of the analysed animals including the L- strand and the H- strand.

		*Caenorhabditis elegans*		*Daphnia magna*		*Drosophila melanogaster*		*Latimeria chalumnae*		*Danio rerio*		*Ambystoma mexicanum*		*Crocodylus porosus*		*Gallus gallus*		*Mus musculus*		*Canis lupus familiaris*		*Pan troglodytes ellioti*		*Homo sapiens*	
	Strand	L	H	L	H	L	H	L	H	L	H	L	H	L	H	L	H	L	H	L	H	L	H	L	H
Genomic region																									
***TRNF***																				2	5				
***12s rRNA***		**2**	**2**	**6**	**5**	**5**	**5**	**17**	**31**	**17**	**14**	**18**	**37**	**27**	**61**	**17**		**10**	**11**	**10**	**32**	**17**	**43**	**15**	**36**
***16s rRNA***			**8**	**11**	**12**	**5**		**15**	**37**	**30**	**48**	**13**	**26**	**42**	**62**	**29**	**25**	**18**	**29**	**9**	**28**				
***TRNV***								2		4															
***TRNL1***			4				6										5			2					
***ND1***		**2**	**5**	**4**	**18**		**4**	**17**	**20**	**14**	**21**	**3**	**10**	**23**	**58**	**22**	**29**	**14**	**15**	**15**	**27**	**14**	**34**	**25**	**37**
***TRNI***										2	2	2		2											
***TRNM***									3	2	3					2	3		3	2		2	3	2	3
***ND2***			**2**	**2**	**4**		**2**	**2**	**21**	**6**	**22**		**6**	**13**	**13**	**7**	**16**	**3**	**6**	**4**	**15**	**4**	**25**	**8**	**27**
***TRNW***								2										2	3						
***TRNN***								2																	
***TRNC***														3				2							
***TRNY***								3			18		3		3		3								
***COX1***		**2**	**5**	**12**	**29**	**2**		**14**	**41**	**6**	**21**	**19**	**37**	**22**	**41**	**17**	**32**	**10**	**16**	**19**	**20**	**28**	**16**	**36**	**16**
***TRNS1***											5				3		3								
***TRND***														4											
***COX2***				**8**	**10**			**6**	**9**	**11**	**17**	**2**	**9**	**6**	**9**	**15**	**15**		**5**	**4**	**6**	**15**	**12**	**18**	**10**
***TRNK***													4												
***ATP8***																						2	2		
***ATP6***				**7**	**21**		**3**	**11**	**6**	**5**	**7**		**13**	**8**	**23**	**2**		**4**	**11**	**4**	**9**	**3**	**21**	**7**	**30**
***COX3***				**12**	**18**		**2**	**6**	**11**	**4**	**21**	**5**	**10**	**11**	**22**	**11**	**9**	**2**	**6**	**7**	**14**	**8**	**10**	**9**	**27**
***TRNG***																						3			
***ND3***								**2**	**9**	**4**	**2**	**6**	**2**	**13**	**31**	**5**	**8**		**4**	**3**	**8**	**7**	**9**	**11**	**4**
***TRNR***										3							2								
***ND4L***								2	15	2		3	8	4	7		18	2	6			2	6		10
***ND4***				**12**	**23**		**2**	**8**	**17**	**12**	**34**	**8**	**20**	**11**	**35**	**12**	**39**	**7**	**19**	**7**	**23**	**15**	**39**	**13**	**39**
***TRNH***																2									
***TRNS2***											2			4	2		7						4		
***TRNL2***					2									2											
***ND5***					**23**	**2**	**2**	**16**	**57**	**8**	**33**	**2**	**5**	**20**	**78**	**5**	**51**	**14**	**42**	**20**	**64**	**12**	**46**	**16**	**59**
***ND6***								**4**	**18**	**7**	**22**		**12**	**3**	**14**	**9**	**16**		**3**	**2**	**13**		**5**		**5**
**AT-REGION**		6	4																						
***TRNE***																								3	
***CYTB***		**2**	**10**	**5**	**9**	**2**	**2**	**14**	**10**	**16**	**22**	**8**	**13**	**23**	**21**	**13**	**28**	**13**	**11**	**16**	**27**	**20**	**22**	**19**	**33**
***TRNP***														2											
***TRNT***											2														
**D-LOOP**									**3**	**10**	**10**	**9**	**11**	**4**	**9**	**15**	**17**	**9**	**11**	**77**	**26**	**18**	**35**	**14**	**20**
sum of all CpG sites/strand **		14	40	79	174	16	28	143	308	163	328	98	226	247	492	183	326	110	201	192	317	170	332	196	356

* genes in which CpG sites are frequently distributed among species were marked with bold font. ** guanine-cytosine-rich sequences.

**Table 5:** Distribution of CpG sites in regions overlapping more than one gene in mtDNA. *

Species	Strand	Start and Stop of MtDNA Sequence	CpG Count	Genes/Replication Origin Region
***Caenorhabditis*** ***elegans***	L	3341..3356	3	*TRNL1, TRNS1*
***Daphnia magna***	**L**	**1302..1323**	**3**	TRNY *, *COX1*
	**H**	**1293..1319**	**4**	TRNY *, *COX1*
***Latimeria chalumnae***	L	2762..2788	4	*16s rRNA, TRNL1, ND1*
	H	1106..1134	4	*TRNV, 16s rRNA*
	H	2693..2819	12	*16s rRNA, TRNL1, ND1*
	**H**	**5279..5466**	**14**	*TRNN* *, *TRNC* *, *TRNY* *
	H	7857..7908	6	*COX2, TRNK*
	H	8526..8861	25	*ATP6, COX3*
	H	15,468...15,523	6	*CYTB, TRNW*
***Danio rerio***	**L**	**6225..6412**	**14**	*TRNN* *, *rep_origin* *, *TRNY* *
	L	11,558..11,579	3	*ND4L, ND4*
	H	951..1402	36	*TRNI, 12s rRNA*
	H	3727..3873	12	*TRNL1, ND1*
	**H**	**6219..6414**	**18**	*rep_origin* *, *TRNY* *
	H	8802..8845	4	*COX2, TRNK*
	H	9538..9829	23	*ATP6, COX3*
	H	10,883..11,253	26	*ND3, TRNR, ND4L*
***Ambystoma mexicanum***	**L**	**5153..5198**	**5**	***rep_origin*** *
	L	15,333..15,346 15,446..15,463	2 2	*intergenic region*
	H	2606..2649	5	*16s rRNA, TRNL1*
	**H**	**5051..5179**	**10**	*TRNA* *, *TRNN* *, *rep_origin* *
	H	15,336..15,355 15,439..15,464	3 3	*intergenic region*
***Crocodylus porosus***	L	11,619..11,679	4	*TRNS2, intergenic region*
	L	13,688..13,713	4	*ND5, ND6* *
	H	3624..3720	11	*ND1, TRNI*
	H	4664..5023	24	*ND2, TRNW*
	H	7648..7931	21	*COX2, TRNK*
	H	9918..9935	2	*TRNR, ND4L*
	H	11,590..11,617	4	*intergenic region*
	H	11,822..12,011	15	*TRNL2, ND5*
***Gallus gallus***	H	1199..2726	98	*D-loop, TRNP, 12s rRNA, 16s rRNA, TRNV*
	H	4971..5040	8	*ND1, TRNI*
	H	6404..6523	10	*TRNA* *, TRNN *
	H	9542..10,097	37	*ATP6, COX3*
***Mus musculus***	**L**	**5167..5187**	**3**	***rep_origin***
	**H**	**5168..5186**	**18**	***rep_origin***
***Canis lupus familiaris***	**L**	**5187..5226**	**7**	*rep_origin* *, *TRNC* *
	L	7969..7991	3	*ATP8, ATP6*
	H	2652..2692	5	*16s rRNA, TRNL1*
	H	4983..5183	12	*TRNW, TRNA* *, *TRNN* *, *rep_origin* *, *TRNC* *
	H	7982..7995	3	*ATP8, ATP6*
***Pan troglodytes ellioti***	L	5156..5187	4	*intergenic region, TRNC* *
	L	7951..8003	5	*ATP8, ATP6*
	**H**	**4946..5783**	**57**	*TRNW, TRNA* *, *TRNN* *, *TRNC* *, *TRNY* *, *COX1*
	H	7964..8004	6	*ATP8, ATP6*
	H	8558..8720	12	*ATP6, COX3*
***Homo sapiens***	L	5737..5768	5	*intergenic region, TRNC* *
	**H**	**5540..6268**	**50**	*TRNW, TRNA* *, *TRNN* *, *TRNC* *, *TRNY* *, *COX1*

* CpG sites that are frequently repeated in the overlapping replication origin region, tRNA encoding genes, and COX1 gene were marked with bold font (genes encoded on the L-strand).
